# Smart Detecting and Versatile Wearable Electrical Sensing Mediums for Healthcare

**DOI:** 10.3390/s23146586

**Published:** 2023-07-21

**Authors:** Ahsan Ali, Muaz Ashfaq, Aleen Qureshi, Umar Muzammil, Hamna Shaukat, Shaukat Ali, Wael A. Altabey, Mohammad Noori, Sallam A. Kouritem

**Affiliations:** 1 Department of Mechatronics Engineering, University of Wah, Wah Cantonment 47040, Pakistan; ahsan.ali@wecuw.edu.pk (A.A.); uw-21-mts-bsc-007@wecuw.edu.pk (M.A.); uw-21-mts-bsc-016@wecuw.edu.pk (A.Q.); uw-21-mts-bsc-014@wecuw.edu.pk (U.M.); shaukat.ali@wecuw.edu.pk (S.A.); 2 Department of Chemical and Energy Engineering, Pak-Austria Fachhochschule: Institute of Applied Sciences and Technology, Mang 22621, Pakistan; b20f0001che001@fcm3.paf-iast.edu.pk; 3 International Institute for Urban Systems Engineering (IIUSE), Southeast University, Nanjing 210096, China; 4 Department of Mechanical Engineering, Faculty of Engineering, Alexandria University, Alexandria 21544, Egypt; sallam.kouritem@alexu.edu.eg; 5 Department of Mechanical Engineering, California Polytechnic State University, San Luis Obispo, CA 93405, USA; 6 School of Civil Engineering, University of Leeds, Leeds LS2 9JT, UK

**Keywords:** wearable sensors, energy harvesting, electrical sensing mediums, piezoelectric, thermoelectric, electrostatic, healthcare

## Abstract

A rapidly expanding global population and a sizeable portion of it that is aging are the main causes of the significant increase in healthcare costs. Healthcare in terms of monitoring systems is undergoing radical changes, making it possible to gauge or monitor the health conditions of people constantly, while also removing some minor possibilities of going to the hospital. The development of automated devices that are either attached to organs or the skin, continually monitoring human activity, has been made feasible by advancements in sensor technologies, embedded systems, wireless communication technologies, nanotechnologies, and miniaturization being ultra-thin, lightweight, highly flexible, and stretchable. Wearable sensors track physiological signs together with other symptoms such as respiration, pulse, and gait pattern, etc., to spot unusual or unexpected events. Help may therefore be provided when it is required. In this study, wearable sensor-based activity-monitoring systems for people are reviewed, along with the problems that need to be overcome. In this review, we have shown smart detecting and versatile wearable electrical sensing mediums in healthcare. We have compiled piezoelectric-, electrostatic-, and thermoelectric-based wearable sensors and their working mechanisms, along with their principles, while keeping in view the different medical and healthcare conditions and a discussion on the application of these biosensors in human health. A comparison is also made between the three types of wearable energy-harvesting sensors: piezoelectric-, electrostatic-, and thermoelectric-based on their output performance. Finally, we provide a future outlook on the current challenges and opportunities.

## 1. Introduction

In recent years, wearable healthcare devices have brought an exponential change in human life. The different versatile sensors follow the thermoelectric, piezoelectric, and electrostatic principles, which are combined using materials that are flexible and easy to handle by users [1,2,3,4,5]. This idea and concept have picked up pace rapidly and many products are introduced into the commercial market related to wearable healthcare monitoring (WHM), some of which are health bands, Apple Watches, Fitbit, Withing, Oura Ring, ECG Patch Monitors, and so on [6,7]. These all are wearable healthcare products available in the market and are based on different mediums and sensors, i.e., pressure sensors, temperature sensors, and optical sensors, etc. Here in this review, we describe these electrical sensing mediums, which include three main piezoelectric-, electrostatics-, and thermoelectric-based wearable sensors for healthcare monitoring. Although all these products are somewhat of a breakthrough in the health monitoring field, they are still, however, constrained due to some other requirements, such as integration or attachment with human skin, the measurement of minute physiological parameters, or a lack of power generation for their prolonged charging. A key factor in their lacking is the charging of these products, which is somewhat traditional, like the plug and charge process [8]. To overcome these problems or hurdles, multi-functional, stretchable, flexible, self-charging health-monitoring devices are a much-needed necessity [9]. In these health-monitoring devices, their main body comprises multiple types of sensors, which will serve several purposes, such as the power generation of the product and the detection or monitoring of the physiological movements and signals in the human body, such as the temperature of the body, blood glucose, heart rate, fever, heart rhythms, blood oxygen, sleep patterns, cold checks, and acceleration, etc. [10,11]. To achieve these features or purposes, we must see the different sensors fabricated through multiple mechanisms, such as single or hybrid mechanisms [12], and these sensors are divided according to their specific area of expertise [13,14]. For accurate human health monitoring, these sensors should be able to attach to the human skin as effortlessly as possible and the sensing range of these monitoring sensors should be wide, that is, for detecting subtle reactions such as heart rate, pulse, and respiration to vigorous experiences, which include running, bending, and stretching, etc. [15,16,17,18]. Another crucial factor in health-monitoring tools is their sensitivity, which can be further enhanced through better structuring and fabrication [19,20]. The charging or power generation of wearable sensors is also another milestone to go, because, in traditional health-monitoring devices, these products need an external power source for the power generation for its health-monitoring parameters to work, which has multiple effects on the environment, as well as the overall structure of the product [21]. Thus, another reason for using multiple sensors (piezoelectric, thermoelectric, and electrostatic sensors) is to solve these environmental and structure-related problems, and also to achieve the compactness of this health-monitoring device [22,23,24,25]. Electrostatic sensors are versatile and cost-effective tools that have been used for monitoring various processes and clinical environments over the past 30 years [26]. They operate on the principle of static electricity generated by friction between two objects or the triboelectric effect [27]. Electrostatic sensors use an electrode and an insulator to detect the motion of an object. They provide solutions to measurement issues and offer a cost-effective alternative to other types of sensors [28,29,30,31]. These sensors can detect the triboelectric effect, which prompts a surge or electric current that can be used to generate power [32]. In addition to gas turbine engines, electrostatic sensors are used in healthcare for sensing different body movements, including wearable sensors for monitoring blood pressure and blood oxygen levels [33,34,35,36]. Electrostatic sensors are commonly referred to as battery sensors or inductive sensors, which were originally used to track human activity [37,38,39].

In the field of wearable healthcare, wearable electrical sensing mediums such as piezoelectric-, electrostatic-, and thermoelectric-based wearable sensors are regarded as smart and versatile technology. These sensors have a variety of qualities that contribute to their adaptability. First of all, these sensors have the ability to measure several signals or characteristics simultaneously. For instance, strain or mechanical pressure can be detected using piezoelectric sensors, which are able to transform these into electrical signals. They are able to measure a variety of bodily motions, such as muscle contractions, joint motions, and even heartbeat, because of this ability. On the other hand, electrostatic sensors are able to detect changes in charge distribution or electric potential, allowing them to capture tiny movements such as eye blinks and gaze directions. Thermal patterns in the body can be studied and monitored with thermoelectric sensors, which can detect temperature gradients or variations. These sensors’ ability to measure numerous signals broadens the range of the healthcare applications in which they can be used. Second, wearable electrical sensing mediums are appropriate for people of all ages, sizes, and health problems. Sensors are designed to be versatile, flexible, and conformable to various types of bodies. This feature guarantees a secure fit and reliable signal acquisition. Sensors can also be incorporated into a variety of wearable form factors, including clothing, accessories, or patches, making them appropriate for various health problems and monitoring. These sensors’ versatility enables customized and unique heath-monitoring solutions. Healthcare monitoring is made simple and flexible by wearable electrical sensing technologies such as piezoelectric, electrostatic, and thermoelectric sensors. They are useful tools for individualized and ongoing healthcare monitoring because of their capacity to measure many signals, versatility to various body types, and non-invasiveness. By facilitating early detection, personalized treatments, and enhanced general wellbeing, these devices have the potential to fundamentally alter our ability to manage and monitor our health [37,38,39,40].

This article will shed some light on the different wearable smart health-monitoring devices for sensor technology, focusing on the concepts of piezoelectric, thermoelectric, and electrostatic principles, and mechanisms that target the integration of multiple principles. The sensor materials and their integration with the human body are discussed, while keeping in view the different medical and healthcare conditions faced by the human body, that is, shown by the appropriate tables and figures. A performance comparison between these three wearable sensors is also described, along with their applications. Finally, the future outlook and potential of these wearable devices are discussed.

## 2. Foundational Principles

### 2.1. Piezoelectric Principle

To assess several physical characteristics, including body motions, heart rate, and respiration rate, piezoelectric sensors are frequently employed in wearable smart health-monitoring systems (WHMS). A piezoelectric sensor functions primarily by producing an electrical charge in reaction to mechanical pressure or stress [41]. This sensor is made of piezoelectric components, such as quartz or ceramics [42,43]. A piezoelectric sensor in a wearable smart health-monitoring system is often built into a tiny module that is fastened to the body. Mechanical stress is applied to the piezoelectric sensor as the body moves, which causes it to produce an electrical charge [44]. This charge is inversely proportional to the stress or pressure placed on the sensor, which can be used to measure the desired physical parameter.

To respond to external impetuses or stimuli and then propagate them into output signals, a wearable monitoring device generally comprises a sensor, electrodes, and a flexible support substrate [45]. The opted sensor is linked to the electrodes and placed on the surface of the substrate film. As a result, the characteristics and capabilities of the internal sensor dictate the kinds of physical or anatomic signals that come under detection. Blood pressure, heart rate, artery pulse waves, and body motion are among the variables that are constantly monitored, according to the principles of piezoelectricity, [46,47]. The conductivity of the electrodes, which is essential for the flow of electrons during the data transmission and processing, determines the monitoring device’s sensitivity and response time. The use of manufactured devices in healthcare is influenced by substrates, because they act as supporting materials for sensors that enable health status monitoring.

**Figure 1:**
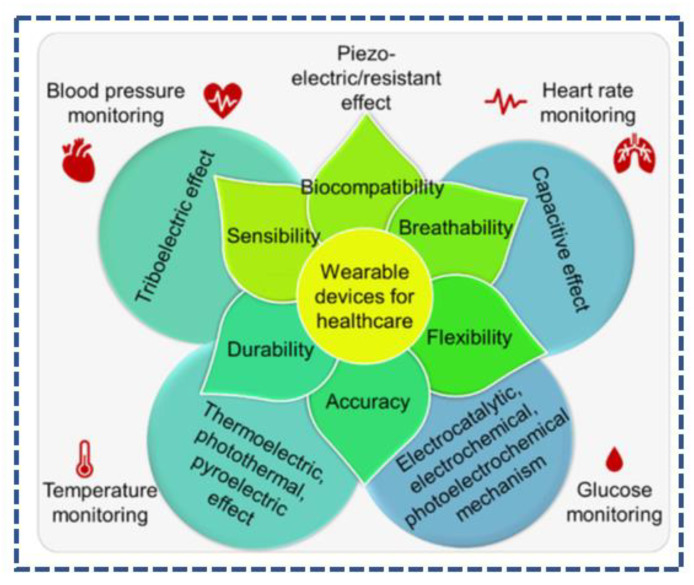
Piezoelectric wearable device in healthcare [48].

In Figure 1, a chart is given, which gives us an overview of different medical condition and then the effects/principles related to these said conditions, as well as some of their properties. It shows us that the piezoelectric principal touches almost all the other effects [48].

**Necessary Elements for Wearable Healthcare Monitoring Devices:** When creating a health-monitoring device, several variables must be considered. The necessity that the electrodes, sensor technology, and supportive substrate do not interact negatively with or harm organs and tissues is known as biocompatibility, and it is an important factor to consider when choosing materials. Second, comfort is always linked to breathing ability when ongoing health monitoring is involved. To release the perspiration produced by the human body and prevent irritation of the skin and tissues, health-monitoring gadgets with porous designs and hydrophilia are greatly wanted. Third, the degree of stretchability and flexibility decides whether the produced device may be included in wearable clothing or patches, etc. Therefore, flexible organic polymers with strong mechanical properties are appropriate options for substrate films. Fourth, the sensitivity and accuracy indicate the reaction time, detection threshold, and distinction between the responding signals and their actual physiological counterparts, all of which have a significant impact on the practical application in medicine. Fifth, the factors of stability and durability are crucial for industrialization and commercialization. Health-monitoring gadgets’ capacity for self-power and self-healing are also two essential elements for their actual use.

### 2.2. Electrostatic Principle

**Figure 2:**
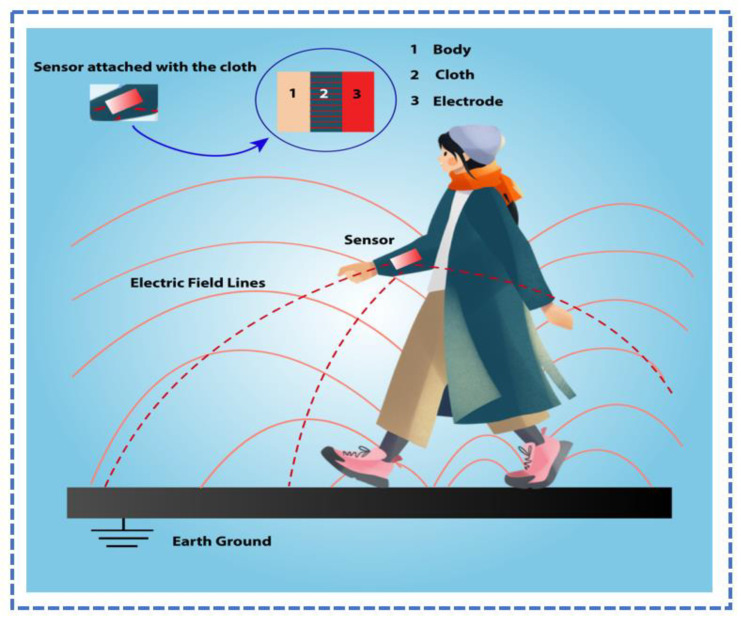
The human body, the sensor, and the Earth’s ground are interconnected through capacitive coupling.

Capacitive interactions arise on human bodies, the electrodes that contact them, and the Earth’s ground whenever an electromagnetic detector is positioned on the hand with a cloth over the electrode and the layer of skin, as shown in Figure 2. An analogous circuitry concept made up of aggregated capacitors may be used to describe the detecting network. The Earth’s ground is capacitive and associated with the human body. An electromagnetic biosensor is powered by a source of energy, and the electrode that holds it is kept above the current potential of the ground. Capacitively connected to the ground’s surface are the conducting electrode plus the immediate Earth plane. The variance in potential across the human body and the point of contact is measured by the electrostatic device [49].

The difference in capacitance across the body and the electrode that contacts it is maintained as constant once the electrostatic probe is strongly connected to the human body. Variations between the charge and linking impedance cause fluctuations in the potential difference between the human body and the surface of the electrode. Because everyday actions have little effect on gross charges, it is conceivable to explain the variations in the potential distinction between the human system and electrode according to fluctuations in body inductance and electrode inductance. Body capacitance shifts with motion, but electrode capacitance can stay unchanged or fluctuate subject to whether the sensing device is linked to a fixed portion of the human body. Dining at an elevated surface, for instance, has little effect on the electrode capacitance within an ankle-worn detector, since that part of the leg is completely motionless. Irrespective of where the sensing device is located on the subject, every movement of the body causes shifts in the potential difference between the skin and the electrode [50,51,52].

### 2.3. Thermoelectric Principle

The cardinal principle of the thermoelectric concept is generating power through temperature change, which can generally be achieved by using two different plates (preferably metals) and joining them at the ends; then, the heat difference at their junctions will give rise to a voltage that is in proportion with the temperature at those connection points [53]. Furthermore, when a current or some charge passes through a thermocouple, it changes the temperature at the junctions of the plates, increasing the temperature of one plate and decreasing the temperature of the other plate, where the rate of heat transfer is proportional to the current. The materials used also have a crucial role in this process. Some recently discovered materials, such as Bi2Te3, and some flexible nanogenerators are used in thermoelectric sensors [54,55]. The most easily accessible source of energy in nature is thermo-energy, and in healthcare, the skin is seen as a feasible source of thermal resistance, which opens up a vast field for thermoelectric sensors. The principle of thermoelectricity rests heavily on the nature of these materials. Some common-use materials in thermoelectric sensors are those such as Bi2Te3-based inorganic materials, along with their alloys and some recently discovered nanocomposites [56,57,58]. Thermoelectric sensors sense external temperature changes, and they are also able to sense external pressure. This was shown in a study, in which it was proposed that a thermoelectric pressure sensor could attain high levels of sensitivity if it was in a coordinated structure with a fractured microstructure [59]. Wearable thermoelectric sensors (TESs) have enjoyed huge amounts of progress in their progressive years, but they still face some complications, some of which are the consideration of heat sinks and the accurate simulation of human thermoregulatory models.

**Figure 3:**
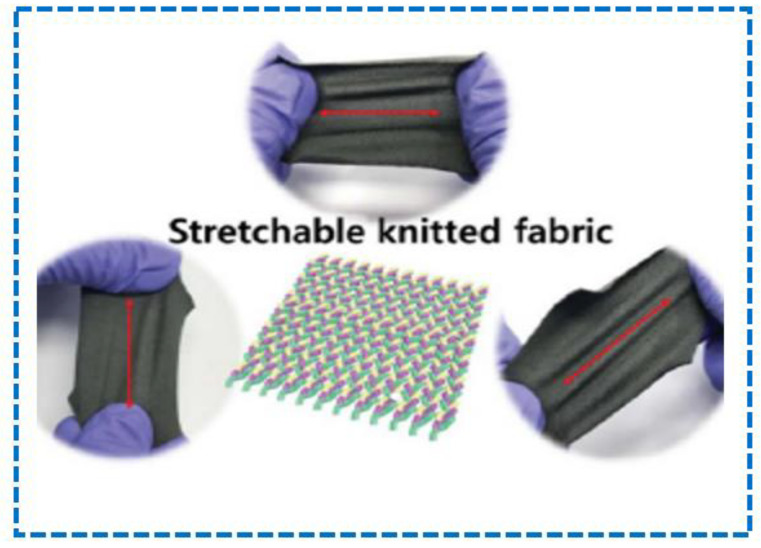
E-skin Stretchable Knitted Fabric [60].

Figure 3 shows a sensor fabricated based on textiles that are processed with some corporate thermos-electric inks with a temperature-sensing ability, which depends on the direction in which they are stretched. This was carried out with the benefits of some fabrics in mind, such as their flexibility, convenience, slimness, adaptability, and simplicity of production, which are all made possible by thermo-electric sensors [60].

Table 1 shows a comparison between different working mechanisms, i.e., the piezoelectric, electrostatic, and thermoelectric mechanisms of some of the wearable devices, along with their applications and advantages, while the challenges faced by these wearables are also listed below.

The bandage can produce one of three responses: first, it may wirelessly communicate the information gathered from a cut to a surrounding a computer system, laptop, or cellphone for inspection by the individual receiving treatment or a healthcare professional. Secondly, it may alleviate infection and inflammation at the wound site right away by administering an antibiotic or other medicament stored inside the bandage. Thirdly, a weak electrical field can be applied to the site to encourage tissue growth and hasten healing [68].

## 3. Sensors Technology in Wearable Healthcare

### 3.1. Thermoelectric Based Wearable Health Monitoring Sensors (TWHMS)

Since its birth in 1821 by Seebeck, thermoelectricity has found its home in many applications’ metrology, measuring temperatures, and refrigeration, as well as use in Peltier cells [69]. Now, the thermoelectric effect includes several effects, the Seebeck effect being the first [70]. The Seebeck effect is the discovery of an Estonian physicist, Thomas Johann Seebeck (1821) [71]. Then, the Peltier and Thomason effects are the discoveries of Peltier (1834) and Thomson (1851), respectively. The concept behind thermoelectrics is distinguishable by its name; the temperature difference between the poles of a semiconductor material creates a voltage, which is also known as the Seebeck effect [72]. Now, thermoelectric sensors involve the Seebeck coefficient (S) and the figure of merit (ZT), which plays a vital role in the material selection for a thermoelectric sensor [73]. The driving force behind a thermoelectric generator is visibly the temperature gradient [74]. The same phenomenon is to be exercised with the human body, where the heat released from the human body via the skin will trigger the temperature sensor and power the wearable healthcare monitoring device [75]. Thus, this proves the feasibility of thermoelectric generators as a useful medium in smart healthcare monitoring [76]. The main idea behind the use of TEGs in wearable healthcare is the provision of high output power, as well as ease of wearing, which is their flexibility [77]. Healthcare monitoring devices that use wearable thermoelectrics and are self-driven are used because of the following reasons [78]: there is no need for a battery for continuous power generation (which can be a main factor for their use as wearable healthcare mediums) [79]. Constant power generation will lead to constant monitoring in healthcare systems without any need for periodic data calculations [80]. WTEGs are being used in sensors and electronics for the construction of thermoelectric sensors [81,82]. A WTEG removes a major factor of periodic monitoring and the measuring of data in self-powered smart healthcare systems, which, in turn, remarkably improves the diagnosis process of the healthcare medium [83,84]. WTEGs compared to other sensor mediums in healthcare have an absolute edge because of their small size and soundless lightweight properties. Furthermore, their output performance can also be enhanced, which is also under research. This accounts for their use in human health-monitoring systems [85]. The current materials that are being focused on to construct WTEGs are bulk inorganic thermoelectric materials, because they provide the best-desired outputs at room temperature compared to some other organic and inorganic materials. The interfacing and reaction of the material with the human skin and body also play a role in selecting the material for the thermoelectric sensor medium [43,86]. TEGs also play a beneficial role in wearable healthcare and monitoring, based on the point that they are non-inclusive of chemical products or any mechanical or movable structures during their fabrication or functioning [87]. Additionally, their fabrication falls under different fabrication techniques such as 3D printing, silicon technology, and other technologies [88,89].

Thermoelectric materials and devices are being used to generate electricity from some common heat sources that are natural and human, along with solar energy harvesting [90,91]. Thermoelectric devices integrated and fabricated into wearable gear, along with energy harvesting from the above sources, will boost the existing healthcare and monitoring system and will be a boost to the future TWHMS [92]. The heat from the human body plays a key factor in the production of thermoelectric healthcare sensor mediums, which makes the device wearable and free from the constraints of conventional battery powering and changeups [93]. Thermoelectric generators can be perfect mediums integrated with some other mediums in smart wearable healthcare systems if they perform according to the desired potentials [94]. The advantages of thermoelectric generators are manifold, along with being environmentally friendly and having a high scalability, reliability, and lengthy lifespans [95]. However, their low energy conversion efficiency has limited their potential as a wearable healthcare sensor medium [96]. While thermoelectric generators have shown promising success in space application fields, they have garnered limited success in wearable healthcare systems. The possible reason for this can be traced to the low energy conversion efficiency of these generators [97].

**Figure 4:**
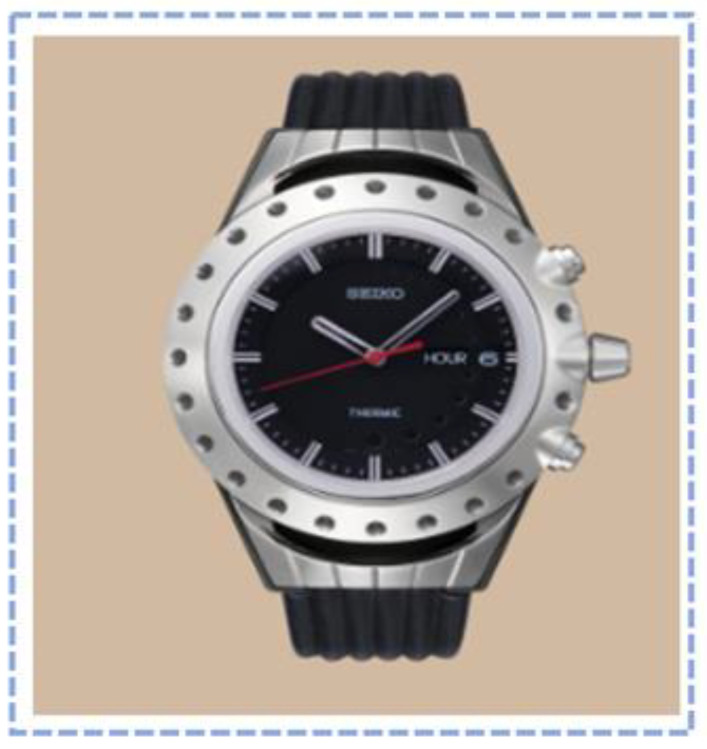
Sieko Thermic Watch [96].

Now, based on the two factors of flexibility and high output power, TEGs (using inorganic bulk materials) are further explained as:

**Rigid Wearable Thermoelectric Generators (r-WTEGS): r-WTEGs**, in general, comprise many Π-type duos of thermoelectric legs connected by metal in series, which are, in turn, combined by two rigid substrates that have a good thermal conductivity [98]. Commercial products use rigid-type technologies because of their better heat collection and dissipation abilities; the reason behind this is the provision of devices with large temperature differences and output power [99]. Rigid Wearable Thermoelectric sensors are in use for a very long time, the Seiko Thermic watch being the first commercial product with a body-heat-powered mechanism, manufactured in 1998 [42], Figure 4. TEGs were further developed for uses such as indoors, for which various designs were created for multiple purposes. In one such design, heat sinks and thermopile pins were used to make the TEG work on a seated individual [96,100].

**Flexible WTEGS:** Considering the intricate and dynamic shaping of the human body, rigid wearable thermoelectric sensors are ruled out of use at some parts. For such situations, the concept and use of flexible TEGs as potential mediums/candidates for wearable smart healthcare sensors have been proposed [101]. This helps to cover the areas such as the arms and dynamic surface parts that are the joints. This has further branched into stretchable and non-stretchable devices based on their areas and modes of working [102].

**Figure 5:**
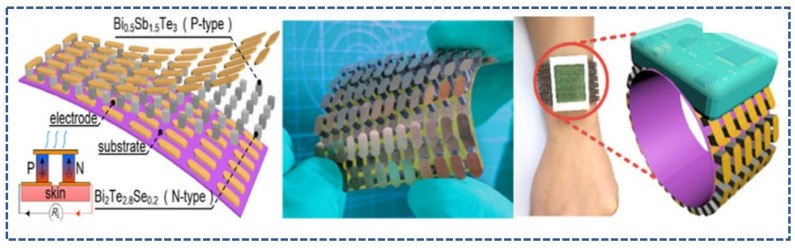
TE Legs connection with flexible electrodes [96].

**Non-stretchable (ns-FWTEGS):** ns-WTEGS have undergone great enhancements in recent times, the reason for this being their wide applications and high efficiency [103]. The simple fabrication of these sensors is a connection of the same thermoelectric legs that are used in r-WTEGS with flexible electrodes, which can be seen in Figure 5. This concludes in an exhibition of excellent flexible, high output, and power density qualities by the ns-FTEG. ns-FETGs are further improved with a combination of different materials, in which some materials are easily oxidized during their operation, while in another design, these problems were rectified but the desired temperature was not attained, and some designs performed excellent, but their fabrication was not suitable for mass production.

**Stretchable FWTEGS:** Stretchable, flexible WTEGSs have sailed well through the areas where the previous categories are lacking; they could be interfaces with complicated and even dynamic surfaces, and they decrease the thermal energy losses in the areas where the previous WTESs are unable to perform [104]. In terms of the type of materials, organic, thin films can be used, but fabrics using inorganic materials have shown promising results. The electrodes in this category are fabricated to be stretchable and liquid metal is used as the material [105]. The devices that use s-FWTEGSs use LM-embedded elastomers layers [106]. SF writers have also shown promising results with snake-shaped copper electrodes [107].

The area of healthcare monitoring has undergone a revolution due to the introduction of wearable, self-powered sensors. By fusing a “flexible thin film thermoelectric generator” that harnesses body heat with a conductive elastomer-based pressure sensor, Wang et al. developed a self-powered wearable pressure-sensing device. Without requiring an external power source, this device permits the continuous tracking of body movements and human physiological signals. In order to monitor temperature changes and capture body heat, wearable temperature sensors that are incorporated with thermoelectric generators have been developed. For the purpose of diagnosing diseases and evaluating one’s health, these sensors allow for both long-term and ongoing temperature monitoring. Self-powered motion and pressure sensors have also been developed, offering an excellent accuracy and flexibility for monitoring physiological indicators, including blood pressure and joint movements. TEGs have been incorporated into e-skin designs to create multifunctional e-skin systems that, among other things, can detect movement, humidity, matter discrimination, and wind flow. The continuous surveillance of cardiovascular activity for the diagnosis and treatment of cardiovascular illnesses is made possible by this. These improvements in wearable self-powered sensors and thermoelectric generator integration have revolutionized healthcare monitoring by enabling the personalized and continuous monitoring of physiological parameters and by enhancing the diagnosis and treatment of a variety of medical problems.

### 3.2. Piezoelectric Based Wearable Health Monitoring Sensor

Globally, heart disease is the leading cause of death [108]. To recognize unusual medical signals and quickly determine the causes of their occurrence, the human body must be accurately and continuously monitored [109]. Wearable technology has recently made a significant contribution to the collection and analysis of health data [110]. For the pre detection, diagnosis, and treatment of diseases, it is a helpful tool [111]. For instance, the ongoing monitoring of physiological signs such as the EKG, heartbeat, blood pressure, and pulse is considered in the analysis and diagnosis of cardiovascular diseases [112]. To address this requirement, several battery-free and autonomous information-collecting methods have been proposed [113]. Many of the currently used methods are cumbersome, costly, and uncomfortable to wear, making them unsuitable for long-term dynamic heart sound monitoring [114]. Heart sound monitoring is another widely used diagnostic method [115]. This method allows for the collection of significant physiological and pathological data related to health [116]. Implantable sensors that generate their own electricity are crucial for patients with special needs and children’s healthy development, as well as for monitoring the health of seniors and individuals who have just recovered from sickness and for providing any necessary medical treatment [117].

**Figure 6:**
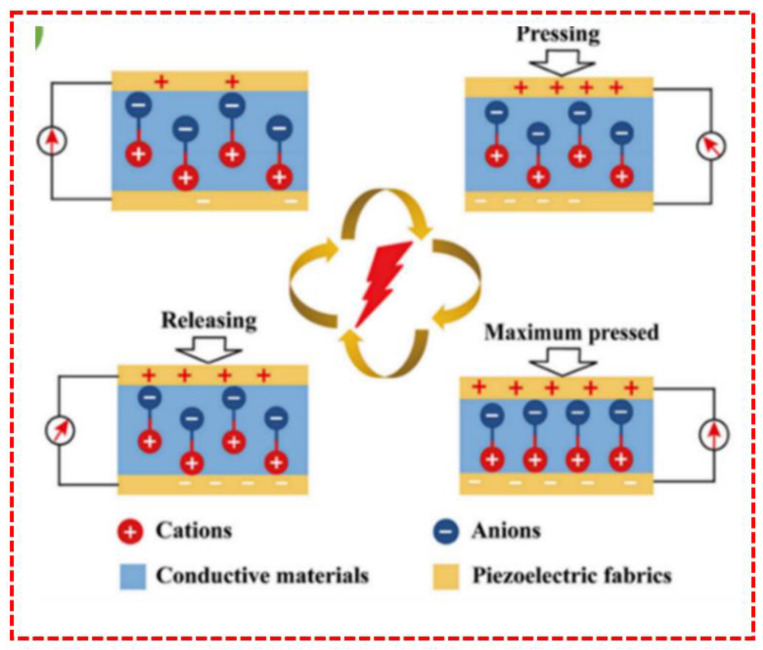
Piezoelectric Principle [123].

Piezoelectric Nanogenerators or PENGs are commonly made up of electrodes and piezoelectric materials [118]. The top and bottom sides of the piezoelectric materials that correspond to these surfaces experience an opposite-sign charge after polarization when PENGs are bent [119]. Figure 6 shows the basic structure of the piezoelectric principle; it is a diagrammatic representation of the procedure through which PENGs generate electricity when their buttons are pressed and subsequently released. The equivalent centers of charge for both cations and anions are still there, even when both types of ions are under stress. Both polarization and the generation of an electric current are nonexistent and do not happen [120]. The volume of the nanogenerator contracts when external pressures are applied, and it stretches oppositely [121]. This has the immediate effect of moving the cation and anion canters of charge, producing electric dipoles, developing a piezoelectric potential between the two electrodes, and creating a new balancing condition [122].

**Figure 7:**
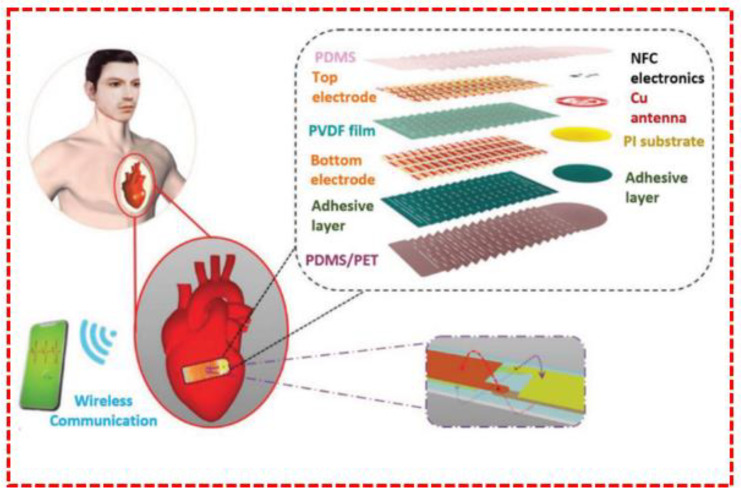
Diagram of the wireless cardiac monitoring systems, kirigami sensing system [124].

For the purpose of detecting small amplitudes of bodily motion, a variety of sensors have been created, with an emphasis on eye movement, artery pressure, heartbeat, and respiration rate. The precision of systems for health monitoring could be increased, and biomedical monitoring could undergo a revolution due to these sensors. Researchers have looked into several materials and technologies for eye motion detection. PZT nanoribbons have been employed to measure eye fatigue by identifying aberrant eyelid movements, However, their toxicity has drawn concern. GaN thin-film-based sensors provide a flexible and biocompatible option for tracking eye movement, with applications in virtual reality and augmented reality, as well as in medical diagnosis. The creation of stretchable, flexible sensors has proved essential for heartbeat monitoring. The real-time tracking of movements and heartbeats is made possible by the high sensitivity and lack of a need for an external power source displayed by nanowire-based sensors. These sensors’ characteristics and stretchability have been improved by kirigami structures made with piezoelectric thin films (shown in Figure 7) [124], enabling the detection of various heart rates and the classification of workout motions. Additionally, wireless real-time arterial pulse monitoring has been made possible using self-powered pulsed sensors made from PZT thin films. The focus of artery-pressure-monitoring sensors is on self-powered systems. PZT thin films have been used to create flexible, self-powered pulse sensors that can detect and produce electrical signals without an external power source. These sensors offer real-time arterial pulse monitoring when worn on the wrist and neck. Diverse methods have been investigated in the case of respiration monitoring, including non-contact mask-based devices, triangular-shaped piezoelectric sensors, and hybrid nanogenerators. These sensors give early health alerts and properly identify breathing patterns. Some sensors, such as earbuds, weather stations, or energy harvesters, even perform numerous tasks. For reliable and portable continuous breath monitoring, low-cost sensors have been created. In general, these sensors have shown the ability to measure and analyze bodily motions, enabling people to monitor their health and assisting with medical diagnostics. They have developed significantly due to the creation of stretchable and biocompatible materials, inventive fabrication methods, and an emphasis on wearability and stretchability.

**Figure 8:**
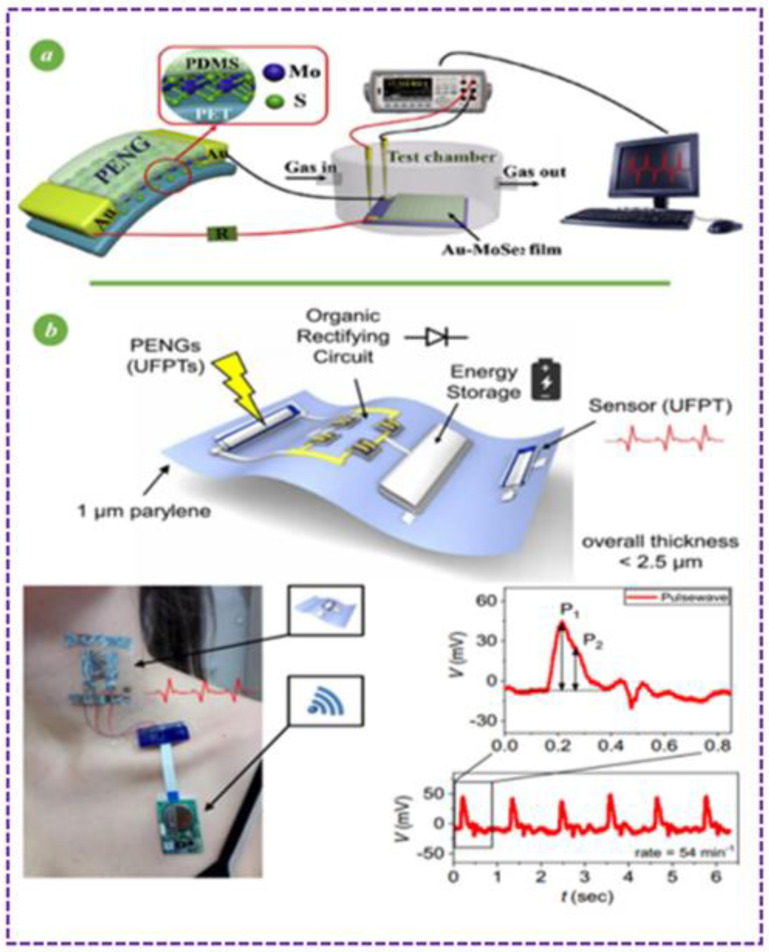
(**a**) Sensor Designs based on materials [129], (**b**) [130].

Piezoelectric Nanogenerators-Based Self-Powered Wearable Sensors: For the first time, an Au-MoSe2 composite ammonia (NH3) sensor was powered at room temperature, using a unique flexible piezoelectric nanogenerator (PENG) based on a two-dimensional (2D) semiconductor MoS2 flake, as shown in Figure 8a. This design also showed a MoS2-based PENG device linked to a person’s body to gather various types of body motion energy, illustrated in Figure 8b, demonstrating the device’s significant potential for usage in wearable electronics [124,125,126,127,128].

In Figure 8, Ferroelectric polymer transducers and organic diodes are shown for unnoticeable sensing and energy-harvesting devices [131]. They have a great flexibility since they are integrated on 1 m thick ultrathin surfaces [132]. Simulations have shown that the sensitivity of extremely flexible ferroelectric polymer transducers is considerably increased by using an ultrathin substrate, which permits mounting them on 3D-shaped objects and stacking them in many layers [133]. Due to their enhanced sensitivity to strain and pressure, rapid response, and outstanding mechanical stability, ultra-flexible ferroelectric polymer transducers form invisible wireless e-health patches for precise pulse and blood pressure monitoring [117,134].

A self-powered PWHMS is demonstrated [135]. Smart sensors are useful because they are easily and affordably manufactured, wearable, long-lasting, responsive, resistant to water and other factors, and versatile enough to be utilized anytime, anywhere [136]. The creation of PENGs represents a significant advance in the field of wearable device self-sufficient sensor technology [137]. In the last few years, wearable, self-powered sensors have achieved significant advancements in the fields of healthcare and medicine [138]. In contrast to traditional self-powered wearable sensors that are powered by an external battery, flexible sensors that are driven by an internal battery have the potential to successfully overcome the limitations of a battery that has a finite lifetime [139]. Moreover, PENGs can remove the uncertainty related to energy conversion in specific locations [140].

### 3.3. Electrostatic Based Wearable Health Monitoring Sensors (EWHMS)

Several industrial processes, mechanical systems, and clinical environments have been continuously monitored and measured using electrostatic sensors over the past three decades [141,142,143]. A wide range of installation conditions can be met by electrostatic sensors due to their cost-effectiveness and versatility. In addition to providing solutions to measurement issues, they provide cost-effective alternatives to other sensors, i.e., acoustic, capacitive, optical, and electromagnetic sensors. Although electrostatic sensors already have a wide range of applications, their underlying sensing principle and subsequent system characteristics are usually the same.

**Figure 9:**
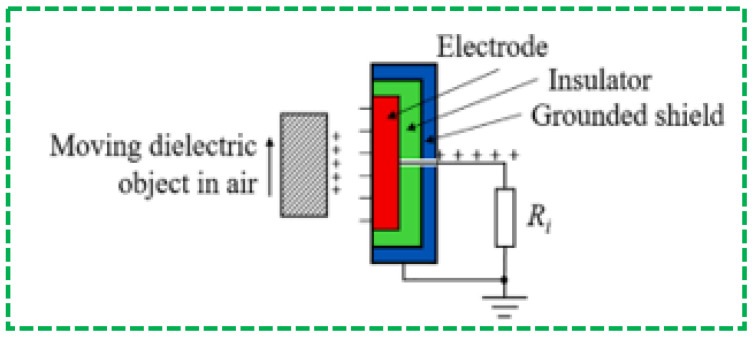
The sensing principle of an exposed electrode for detecting a non-conducting object in motion through electrostatic induction [146].

The basic phenomenon of electrostatic sensors can be understood through daily life examples, as it can be present anywhere. An electric shock occurs when you touch a metallic doorknob after walking over a plastic floor. There may have been times when you felt a tiny electric spark in your hair when you removed a woolen jumper. Considering such a common phenomenon, it can be concluded that static electricity is a result of friction between two objects [144,145]. Static electricity will be generated as soon as two objects are in close physical contact through rubbing. Electrostatic charges can also appear between two objects that have been in close contact and then separated without physical friction. All materials are made of atoms and atoms are made of electrons, protons, and neutrons. When two different materials are rubbed together, then one of them might gain more electrons from the other material, and this process is called the triboelectric effect. This effect occurs daily in our lives and electrostatic sensors can be used to detect it. Figure 9 shows the simple use of electrostatic sensors, using an electrode and insulator for detecting the motion of an object.

Electrostatic sensors are also used in healthcare for sensing different body movements; some of these are ambient and others are wearable. Embedded within everyday living spaces, ambient sensors quietly perceive alterations in the surroundings resulting from human actions, such as temperature, light, sound, pressure, and others. Ambient assisted living systems leverage these sensors to track the welfare of individuals in their living environments. Despite being the most prevalent form of ambient sensors, video cameras give rise to significant privacy and security apprehensions [147].

In contrast, the limitations mentioned above can be addressed by using wearable sensors affixed to different areas of the human body. Accelerometers and gyroscopes, which are inertial sensors, are commonly used for detecting and monitoring movement. Magnetometers are often utilized in conjunction with inertial sensors to enhance motion tracking. Previous research has investigated the monitoring of human activity through electrostatic sensors. Various terms, such as electric potential sensors and capacitive sensors, have been employed in prior publications to refer to the same approach.

According to (Lee, K. H., 2021), researchers from over the globe developed a wearable electrostatic sensor that could monitor blood pressure non-invasively. The device was found to be accurate and reliable and could be used for the long-term monitoring of blood pressure in patients with hypertension. Similarly, according to (Dey, S. et al., 2015), a development in wearable electrostatic sensors for monitoring blood oxygen levels was performed. The sensor was found to be highly sensitive and accurate and could potentially be used for monitoring patients with respiratory disorders. According to (Wang, J. et al., 2014), another promising application of electrostatic sensors in healthcare is in the monitoring of the glucose levels in patients with diabetes. In a study published in the journal *Sensors and Actuators B: Chemical*, researchers from the University of California, San Diego, developed a wearable electrostatic sensor that could monitor the glucose levels in sweat. The device was found to be accurate and could potentially be used as an alternative to traditional blood-glucose-monitoring methods.

A unique wearable electrostatic sensor that can track the torso and limb movements throughout daily activities from any point on the body was proposed by Y. Hu and Y. Yan in 2022. A charge amplifier is used by the sensor to transform the potential electrical difference between the body’s surface and the electrode into a voltage signal. Amplification, digitization, filtering, and ZigBee transmission follow this signal. This sensor’s capacity to record movements from various body regions has been proven by experimental evaluations. There is a lot of potential for biological and healthcare applications using this wearable sensor. A transparent and adaptable electrostatic sensing interface for active eye tracking (AET) devices was presented by Shi et al. To increase the capacitance and charge storage capacity, this interface uses the structure of a triple layer, which includes a di-electric bi-layer and silver nanowire electrode layer. An accurate oculogyric detection with a high angular resolution is made possible by the interface’s outstanding electrostatic charge distribution and charge-keeping rate. As a result of the AET system’s ability to decode eye movements in real time, new possibilities for applications, including user preference recording, eye-controlled HCI (human–computer interaction), VR, and medical monitoring, are now possible.

Overall, electrostatic sensors have a lot of potential for the development of innovative wearable devices for healthcare monitoring. With further research and development, a hand band watch that can monitor different health clinical conditions, such as blood pressure and blood oxygen levels, could become a reality.

Table 2 shows the properties and areas of action of some of the sensors used for healthcare monitoring, and their characteristics and implementations.

## 4. Performance Comparison among Wearable Energy Harvesters

Piezoelectric wearable energy harvesters have the benefit of being able to produce high voltages, and their manufacturing processes could allow for further miniaturization. These devices are useful for a variety of applications, because they can effectively transform mechanical vibrations into electrical energy. They can be integrated into wearable devices due to their small size and high voltage output, which makes them suitable with many different electronic devices. Additionally, improvements in their manufacturing methods may result in PWHMSs that are even smaller and more effective. EWHMSs have a relatively simple fabrication method and allow flexibility with Micro-Electro-Mechanical Systems technology. They can produce large voltage levels and efficient low-frequency signal capture. Electrostatic harvesters need a small space between the plates to generate high power densities, however, this might cause the dielectric material to deteriorate with time. To begin energy generation, these devices need an initial voltage, which makes the system more complex. Despite these drawbacks, EWHMSs have demonstrated promise in absorbing energy from different kinds of motion. The integration of TWHMSs is quite simple. Depending on what temperature the device has, they can yield significant power densities. However, a method is needed to maintain this temperature difference, which would need the use of more power. Thermoelectric harvesters typically produce less energy and less voltage output than piezoelectric and electrostatic harvesters. However, they have the advantage of producing electricity from temperature gradients, making them appropriate for applications where there are substantial temperature changes.

As a result, piezoelectric WEHs have the potential for further miniaturization and offer high voltage outputs. Electrostatic WEHs require a small gap and starter voltage, but are easy to fabricate and are adaptable with MEMS technology. Thermoelectric EHs have a lower energy production and output voltage requirements, but they are simple to incorporate and can generate large power densities based on temperature changes. The choice is based on the application requirements and environmental factors, as each technology has advantages and disadvantages of its own. Table 3 shows a comparison of these piezoelectric, electrostatic, and thermoelectric wearable energy harvesters based on their output performances.

## 5. Applications of Wearable Energy Harvesters

Numerous uses for piezoelectric sensors exist, including wearable smart health-monitoring devices. Along with piezoelectric, we can also see electrostatic and thermoelectric applications of the sensors. Below are some of the applications of the smart wearable sensors that are described above.

### 5.1. Heart Rate Monitoring

By picking up on the vibrations that the heartbeat causes, piezoelectric sensors can be used to determine heart rate. These sensors can be included in a wearable device, such as a wristband or chest strap, and used to continuously measure heart rate [190]. While piezoelectric sensors play an important role here, thermoelectric sensors can also be used in the same wearable devices for energy generation, which help in the monitoring of the heart rate for long periods, without the need to change the batteries, hence making them perfectly self-sustaining. This is great step, keeping in sight the concept of pacemakers that are used for abnormal heart monitoring. While this concept somewhat ancient and is in the implantable category, with the integration of piezoelectric and thermoelectric sensors, implantable heart-rate-monitoring technology can be shifted to self-sustaining wearable heart-rate-monitoring devices [191].

### 5.2. Respiration Monitoring

The movement of the chest or abdomen during breathing can be detected using piezoelectric sensors, which can then be used to estimate respiration rate. Monitoring breathing patterns and spotting changes that can point to respiratory issues can be performed using these data [192,193]. The pressure or piezoresistive have shown great potential in this field, in the form of smart chest belts and smart shirts. Now, smart chest belts and shirts are simple, comprising smart piezoresistive sensors that sense the deformity caused due to the movement of the chest that is the result of breathing, and then the difference in the form of smart belts shows us breathing patterns and respiratory results [194].

### 5.3. Movement Tracking

In a wearable device, piezoelectric sensors can be utilized to monitor movement and activity levels. These sensors, for instance, can pick up on limb movements during exercise or the vibration of footsteps [195]. An excellent example of such a principle are the insole devices used in shoes that use piezoelectric sensors, which sense changes in pressure through the steps of humans and can track simple human movements such as walking or running. Movement tracking is best for exercise purposes such as burning calories, through activities such as walking. The small and large deformations caused due to exercise provide the necessary data for health monitoring, which are collected by the pressure and strain sensors that are based on the piezoelectric principle [196].

### 5.4. Sleep Monitoring

Piezoelectric sensors can be used to track sleep patterns by spotting motions and vibrations at odd hours. Using these data, one may monitor their sleep patterns and spot issues such as sleep apnea [197,198].

### 5.5. Fall Detection

By monitoring the force with which a person’s body strikes the ground, piezoelectric sensors can be used to detect falls. In the event of a fall, this information can be used to contact the emergency services or carers. In addition, thermoelectric and electrostatic sensors are important here, because almost all physiological reactions of the human body result in heat and movements of different kinds, which can be sensed by different sensors based on the piezoelectric, electrostatic, and thermoelectric principles and cover the majority of the human body’s reactions. Based on the data collected by the sensors, an appropriate alert system and on-the-spot health measuring and monitoring can be performed, and the appropriate treatment methods can be prepared based on the same data compiled by the sensors’ monitoring.

### 5.6. Smart Bandages

**Figure 10:**
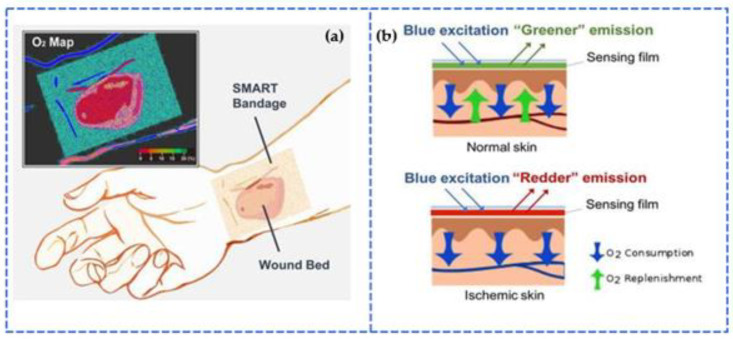
(**a**) O2 Map of Smart Bandage (**b**) Functionality of a Smart Bandage [199].

A new type of smart bandage that uses electrostatic sensors may make treating wounds simpler, more efficient, and less expensive. Despite traditional dressings, which are made of multiple layers of a sponge-like substance, smart bandages are made of a resilient and stretchable material with integrated circuitry and medicine. Electrostatic sensors keep an eye out for chemicals such lactate or uric acid, as well as pH or temperature changes in the wound that could indicate a bacterial infection or inflammation, which are shown in Figure 10a,b [199]. As shown in Figure 10b, a SMART bandage with an oxygen sensor is placed on healthy skin. Because of the high oxygen content in the tissue, the detecting phosphor’s red emission is quenched. The fluorescent reference dye causes the entire film to radiate a green color. Similarly, an oxygen-sensing SMART bandage is applied on ischemic skin at the bottom of the figure. Because there is less oxygen in the tissue, the bright red emissions of the detecting phosphor outperform the reference dye’s green fluorescence. The entire bandage is red in color.

## 6. Mechanisms

The above-discussed principles fall under the single-mechanism category, and they are explained in that capacity. Apart from the single mechanism, there are other mechanisms, namely two-principle and three-principle mechanisms, and these fall under the hybrid mechanism category.

### 6.1. Two-Principle Mechanisms

**Figure 11:**
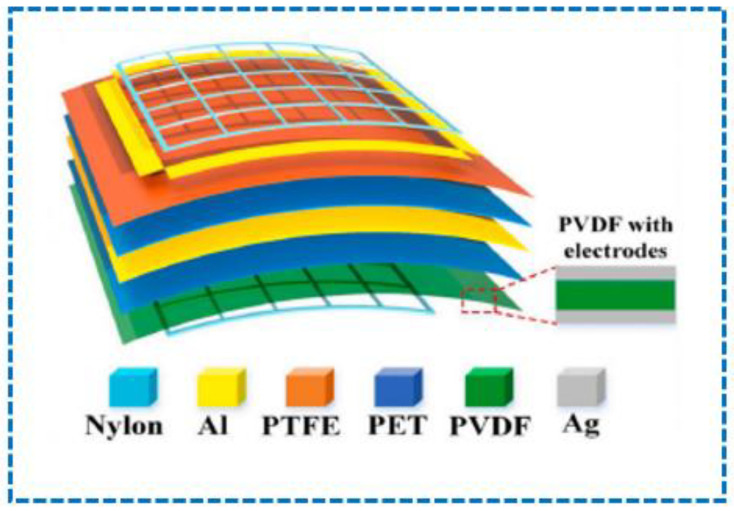
Self-powered piezoelectric and triboelectric sensors which can track vertical force and can also trace the finger [200].

The need for a two-principle mechanism has been raised because the detection of multiple healthcare parameters is not achieved by a one-principle mechanism. Therefore, it is a crucial part of wearable smart health-monitoring systems to have hybrid two-principle mechanisms, because these will cover more health-related physiological parameters compared to single-principle sensors. To fully understand this mechanism, we will see the progress that has been achieved for two-principle mechanisms up until now. In a study, a self-powered sensor was created using triboelectric and piezoelectric principles; hence, a two-principle mechanism was applied. In Figure 11, in the fabricated sensor, the triboelectric principle was applied for finger tracing in the xy-direction and the piezoelectric principle was applied to the check the finger pressure in the z-direction. This is a very versatile concept, as it will have other distinguished applications in other fields such as VR and AR or wearable electronics, etc. [200].

Thus, in another study, a hybrid sensor was created by combining the piezoelectric and resistive principles. The main focus was the proposition of smart skin comprising a PVDF and reduced graphene oxide(rGO) microtome structure; here, the nature of the materials used was majorly kept in view, so the piezoelectric and piezoresistive nature of the PVDF-rGO composites made the fabricated sensor perceive static and dynamic pressure along with temperature [201]. The study in the direction of a combination of piezoelectric and thermo-electric mechanisms was performed using organic piezoelectric poly (vinylidene fluoride) and thermos-electric polyaniline (PANI)-based composite films, for fabricating a flexible sensor that could perceive tactile stimuli and temperature without interference [202]. According to the above-mentioned studies, we come to understand that two-principle mechanisms are much more important and effective in the case of the fabrication of wearable smart health-monitoring commercial products, because it is has been proved to a great extent that, by using two single-principle based sensors, we can achieve multiple targets, such as power generation for making active sensors, as well as the detection of multiple physiological human interactions and signals.

### 6.2. Three-Principle Mechanism

**Figure 12:**
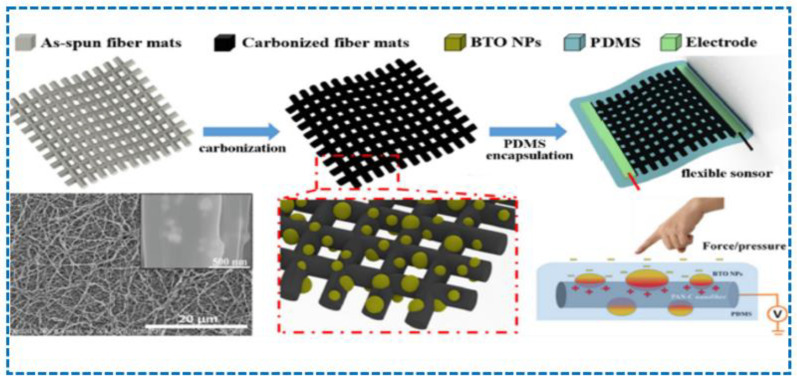
A fabricated sensor based on three-mechanism principles that measure pressure, curvature, and human reactions [203].

The two-principle mechanisms that enjoy an edge over single-principle sensors still fall short compared to three-principle mechanisms, because they are less flexible and their area of working is small compared to three-principle mechanisms, which can be generally understood because three-principle sensors use three sensor principles, which is more than two-principle mechanisms that use two principles. Three-principal mechanisms can be analyzed using the different studies performed to fabricate different sensors using three-principle mechanisms. In a study, it was reported that a carbonized electro spun polyacrylonitrile/barium titanate (PAN-C/BTO) nanofiber film sensor was fabricated using the piezoelectric, piezoresistive, and triboelectric principles; now, the importance of this sensor compared to a sensor that is fabricated using a two-principle mechanism is far more appealing, because this fabricated sensor can measure two parameters, namely pressure and curvature, and this can be performed independently or simultaneously, which is shown in Figure 12. Along with this, the use of titanate nanoparticles (BTO NPs) made its sensitivity very convincing, as it could perceive multiple human reactions such as gait, swallowing, and finger tapping [203].

**Figure 13:**
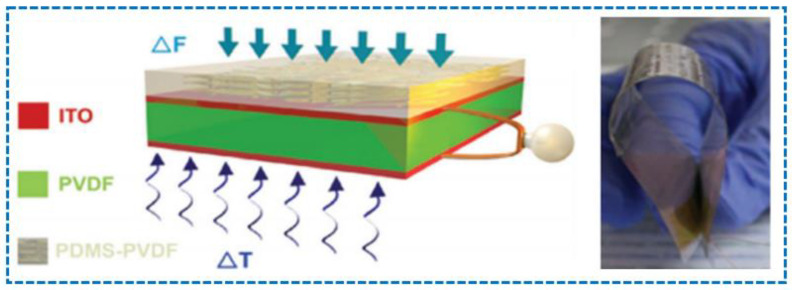
A flexible nanogenerator layered sensor based on piezoelectric, pyroelectric, and triboelectric concepts [204].

In another study, the principles of piezoelectricity, pyroelectricity, and triboelectricity were combined to fabricate a hybrid transparent and flexible nanogenerator. In this sensor, there was a layered structure, and the triboelectric layer used a PVDF nanowires-poly (dimethyl siloxane) (PDMS) composite film, while the other two layers of piezoelectric and pyroelectric parts were made up of polarized PVDF film, and the electrodes were made of indium tin oxide (ITO), as shown in Figure 13 [204].

Thus, in the synopsis of this, we have come to understand that WHMSs have brought a visible ease in human life and the possibilities they can create for human health monitoring are boundless. All of this has been achieved and much more can be achieved using the different ways that these multiple principles and mechanisms can be combined. While the fabrication itself should be less costly, features such as accuracy, conciseness, and flexibility should meet the demand of human healthcare and detection.

## 7. Future Perspective and Conclusions

This study analyzed the literature on wearable devices and sensors for monitoring people’s activities. Human activity monitoring is a thriving research field, and there has been substantial economic growth. The rapid development of microelectronics and wireless communication technologies, in addition to the miniaturization of sensing devices, could account for this improvement. A portion of these cutting-edge wearable sensors are overwhelmed by business wrist-watch sensors, for example, Apple Watch 10, and clinical fixes, for example, the iRhythm Zio patch237 and Abbott’s Free-form patch238, for persistent health surveillance. It is anticipated that a lot more lightweight, high-performing wearables will be able to track a variety of activities.

Future gadgets will also take into consideration the challenges that the present design faces, including inaccuracies, unreliability, high power consumption, limited battery life, and the lack of multi-sensing capabilities. Additionally, the structure of the human body should be considered while designing future wearables. Formal and informal surveys have indicated a growth in interest and the subsequent usage of wearable devices soon, with the cost of the gadgets also being projected to decline, culminating in their widespread application in society.

By 2027, it is anticipated that wearable technologies will save hospital expenses by 16%, and by 2037, remote patient-monitoring gadgets might help save $200 billion. Hospitals can increase their accuracy by keeping track of how many visitors each patient receives and how long they spend in the hospital by having a wearable gadget on hand. The care for patients can be better planned with this information.

## Figures and Tables

**Table 1:** Comparison of the different working mechanism of the wearable devices.

Methods	Advantages	Applications	Degree of Advancement at the Moment	Major Execution Challenges	Ref.
Thermoelectric	Cheap price, no servicing, less weight, superior flexibility and dependability, nothing that moves, simple section, simple to expand	Photo acoustic oximeter, epicardial, audiological assist, ECG, EMG	Versatile TEG device, enhanced by the addition of nanocrystals	Heat variation is necessary, and room temperature transformation of energy is poor	[61,62]
Piezoelectric	The greater voltage at the output, the less space, easy method, extremely delicate to apply tension, a lot of power density	Ventricular pacemaker, pressure gauges, accelerometers, and artificial joints	PEG is extensible and elastic, enhanced by the addition of nanocrystals	Specific supplies are needed, poor performance at low frequency, fragile, high-performance piezoelectric composites, both a small current and elevated impedance	[63,64]
Electrostatic	In harmony with the manufacturing process for MEMS, a tiny scale with an excessive energy density, low-frequency vibration, substantial output power	Neurological stimulator, watch, and ventricular pacer	Sophisticated clothing, electret-based turbines, and polymer-based sources	A different voltage source is required, there must be a mechanical halt, greater voltage, protection is needed, the efficiency is reduced by parasitic elements and current leakage	[65,66,67]

**Table 2:** The general properties of the sensors include active regions on the human body and implementation.

Sensing Material	Sensing Technology	Characteristics	Active Area	Implementation	Ref.
Bi2Te3	FTEG	Flexible	Hands, Arms	This sensor can be used on hands and arms it can sense temperature	[59]
Bismuth Antimony Telluride grains assembled on a flexible Polyimide Film	FTENG	Flexible	Hands	This sensor can be used to measure temperature, humidity, human motion, acceleration, etc.	[96]
PVDF	PENG	Stretchable	Knee	This can be used for only health monitoring	[148]
PVDF-TrFE NWs	PENG	Flexible	Finger, Wrist	This can sense finger movement and sense temperature	[149]
FEP and Au	TENG	Rigid	Ears	These can be used for ears (hearing aids)	[150]
Rubber and Al	TENG	Stretchable	Stomach	This can be used to monitor stomach health (respiration)	[151]
PET and Rubber	TENG	Rigid	Soles (inner)	This can be sued to monitor the gait	[152]
PZT fibers	PENG	Flexible	Heart, Lungs, Diaphragm	This can be used for energy harvesting	[153]

**Table 3:** Comparison of piezoelectric, electrostatic, and thermoelectric wearable energy harvesters based on their output performance.

WEH	Material	Output Current	Power Density	Output Voltage	Output Power	References
Piezoelectric	BaTiO_3_ and P(VDF-TrFE)	4 µA	102.9 µWcm^−3^	14 V	7.2 µW	[154]
	AlN	-	400 µWcm^−3^	0.7 V	0.2 µW	[155]
	ZnO nanowires and PVDF	0.96 μA	0.74 µWcm^−3^	6.9 V	6.624 μW	[156]
	ZnO nanorods	20 nA	-	4 V–8 V	40 nW–80 nW	[157]
	Polymer PVDF	-	0.32 µWcm^−3^	2 V	2 nW	[158]
	ZnO nanowires	-	-	25 mV	3.75 pW	[159]
	Ceramic PZT	-	15.9 mWcm^−3^	2 V	158.8 µW	[160]
	BCTZ nanoparticles, Ag nanowires and PDMS	0.8 μA	-	10 V	8 µW	[161]
	ZnO nanowires	-	102.9 pWcm^−3^	0.6 V	90 pW	[162]
	Fiber composite	-	0.0069 mWcm^−3^	2750 mV	0.011 mW	[163]
	PVDF polymer	-	506.66 mWcm^−3^	-	45.6 mW	[164]
	PZT nanowires	0.0000225 mA	0.200 mWcm^−3^	3000 mV	0.00012 mW	[165]
	PVDF	-	2.18 mWcm^−2^	-	0.87 mW	[166]
	PVDF-NaNbO_3_	0.0044 mA	-	3400 mV	-	[167]
	PVDF nanofibers	0.045 mA	14.28 mWcm^−2^	210,000 mV	2.1 mW	[168]
	Polymer threads	-	0.4 mWcm^−2^	90,000 mV	1.1 mW	[169]
	PVDF	0.00003 mA	0.0104 mWcm^−3^	800 mV	0.024 mW	[170]
	PVDF and polyimide	0.225 mA	3.9 mWcm^−2^	45,000 mV	-	[171]
	ZnO nanowires	0.000107 mA	0.44 mWcm^−2^	2030 mV	-	[172]
	PDMS and ZnO nanorods	0.12 mA	-	170,000 mV	1.1 mW	[173]
	Al wires and PDMS	0.21 mA	2.04 mWcm^−2^	40,000 mV	4 mW	[174]
	PVDF	-	0.3 mWcm^−3^	4949 mV	0.6 mW	[175]
	Ceramic PMNZT	-	22 mWcm^−3^	1400 mV	0.6 mW	[176]
	Copolymers polyethylene-polypropylene	-	-	2500 mV	0.00034 mW	[177]
	PTFE and Al	-	0.125 mWcm^−2^	15,020 mV	0.210 mW	[178]
	PZT-5A	-	0.00059 mWcm^−3^	2470 mV	0.051 mW	[179]
	-	-	0.0074 mWcm^−2^	7680 mV	0.0037 mW	[180]
Electrostatic	Cotton threads, CNT and PTFE	0.00001122 mA	0.0001 mWcm^−2^	-	0.00091 mW	[181]
	TTF-TCNQ	-	0.100 mWcm^−2^	376 mV	6 mW	[182]
	Polyimide and PET films	-	0.0019 mWcm^−2^	0.39 mV	-	[183]
Thermoelectric	CNT/P3HT nanocomposite	-	-	41.8 mV	0.032 mW	[184]
	Bi_0.5_Sb_1.5_Te_3_ and Bi_2_Se_0.3_Te_2.7_	0.0158 mA	0.00014 mWcm^−2^	14.2 mV	0.000224 mW	[185]
	Fabric, Bi_2_Te_3_ and Sb_2_Te_3_	-	3.8 mWcm^−2^	2.9 mV	0.003 mW	[186]
	Bi_2_Te_3_	-	0.0011 mWcm^−3^	25 mV	0.00208 mW	[187]
	Thermal interface material, ceramic plates, Bi_2_Te_3_ and copper sheet	-	0.0285 mWcm^−2^	108 mV	0.285 mW	[188]
	PDMS, aluminum oxide, Bi_2_Te_3_	1.5 mA	0.0061 mWcm^−2^	14,800 mV	-	[189]

## Data Availability

Not applicable.
